# Long-term feasibility of the new sutureless excimer laser-assisted non-occlusive anastomosis clip in a pig model

**DOI:** 10.1007/s00701-020-04533-0

**Published:** 2020-09-02

**Authors:** B. de Boer, T. P. C. van Doormaal, C. A. F. Tulleken, L. Regli, A. van der Zwan

**Affiliations:** 1 Brain Center Rudolph Magnus, Department of Neurosurgery, UMC Utrecht, Heidelberglaan 100, G.03.124, 3584 CX Utrecht, The Netherlands; 2 Brain Technology Institute, Utrecht, The Netherlands; 3 Department of Neurosurgery, Universitätsspital Zürich, Zurich, Switzerland

**Keywords:** Cerebral revascularization, Non-occlusive, Anastomosis, Device

## Abstract

**Background:**

High flow bypass surgery can be a last resort procedure for patients suffering from complex neurovascular pathology. Temporary occlusion of a recipient artery in these patients could result in debilitating neurological deficits. We developed a sutureless, mechanical anastomotic connection device, the SELANA clip (Sutureless Excimer Laser-Assisted Non-occlusive Anastomosis clip: SEcl). In the present study, we aim to determine the long-term non-inferiority of the SEcl technique compared with historical data of the conventional ELANA anastomosis technique.

**Methods:**

A total of 18 SEcl bypasses were created on the carotid artery in a porcine model in 6 different survival groups. Mean application times, flap retrieval rates, hemostasis, patency, flow, endothelialization, and remodeling were assessed.

**Results:**

The mean application time of the SEcl anastomoses was 15.2 ± 9.6 min, which was faster compared with the conventional ELANA anastomoses. The flap retrieval rate of the SEcl anastomoses was 86% (32/37). Direct hemostasis was achieved in 89% (33/37) SEcl anastomoses. Patency in all surviving animals was 94% (17/18). Bypass flow after six months was 156.5 ± 24.7 mL/min. Full endothelialization of the SEcl pins was observed after 3 weeks.

**Conclusion:**

The SEcl technique is not inferior to the ELANA technique regarding patency, flap retrieval rate, flow, and endothelialization. On the basis of a significantly shorter application time and superior hemostasis, the SEcl technique could be preferable over the ELANA technique. A pilot study in patients is a logical next step based on our current results.

## Introduction

**Fig. 1:**
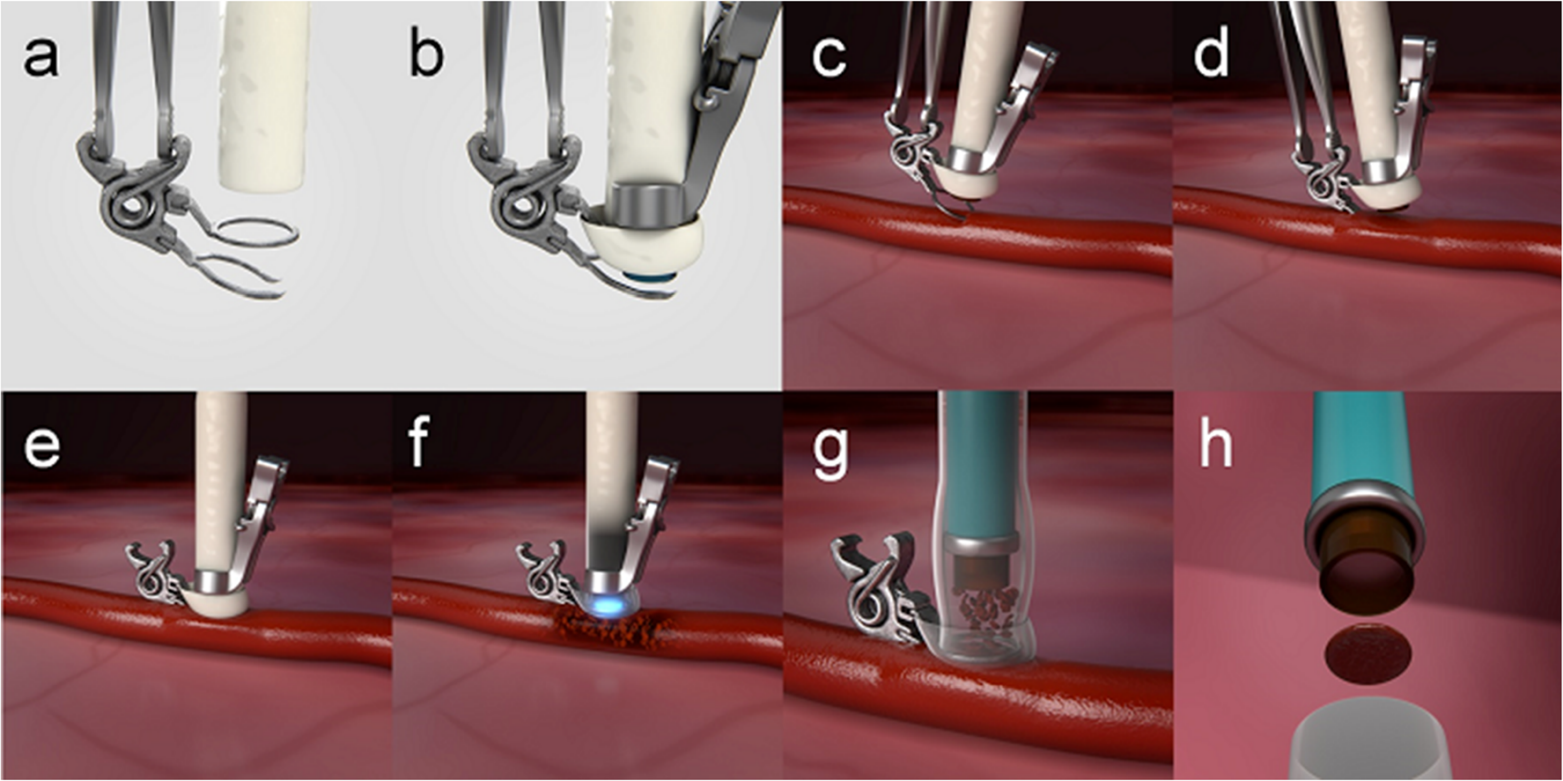
The SEcl technique (adapted from De Boer B, Van Doormaal TP, Stecher D, Redegeld S, Tulleken CA, Regli L, Van der Zwan A (2020) Feasibility of the new sutureless excimer laser-assisted non-occlusive anastomosis clip in a rabbit model. Acta Neurochir 162(1):175–179). (**a**) Introduction of the donor in the SEcl. (**b**) Fixation of the SELANA catheter 2.0 in the donor and SEcl. (**c**) Insertion of the SEcl in the recipient artery. (**d**) Translation, closing, and fixation of the SEcl in the recipient artery. (**e**) The 2 min of vacuum. (**f**) Lasing 3 times at 16.7 mJ. (**g**) Retraction of the SELANA catheter 2.0. (**h**) Flap retrieval

**Fig. 2:**
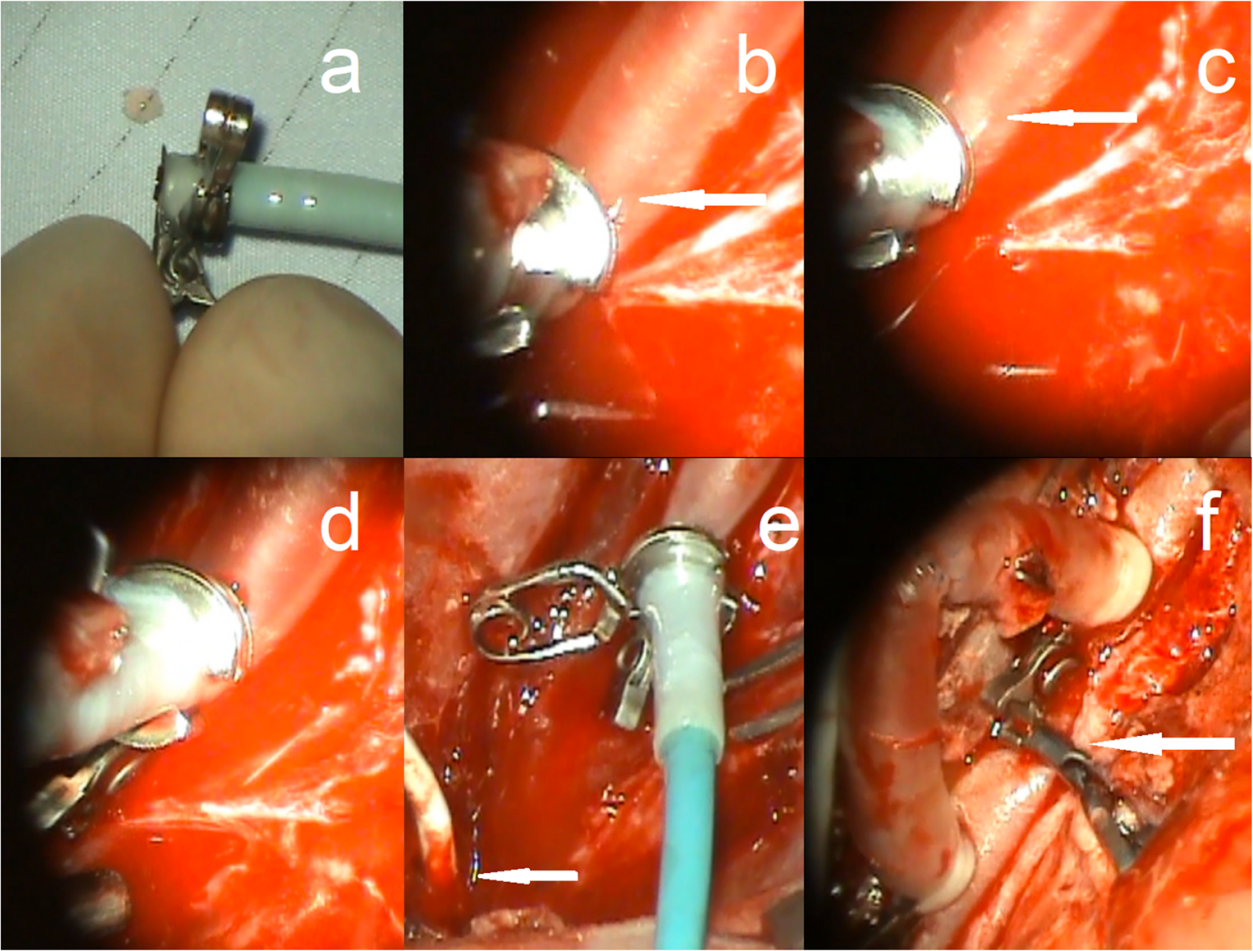
Surgical view of the SEcl technique. (**a**) Fixation of the SELANA catheter 2.0 in the donor and SEcl. (**b**) Insertion of the SEcl in the recipient artery, arrow is pointing at the pins which are clearly visible. (**c**) Translation of the SEcl in the recipient artery, arrow is pointing at the pins which are now in the lumen of the vessel. (**d**) Closing of the SEcl, followed by 2 min of vacuum. (**e**) Lasing 3 times at 16.7 mJ. The arrow is pointed at the single vessel flow meter (Transonic Systems Inc.®, Ithaca, NY, USA). (**f**) Overview of the bypass. The two SEcl anastomoses and one end-to-end anastomosis are all visible. The arrow is pointed to the clip on the carotid artery, which is blocking the flow, and therefore the bypass is now the sole contributor to the anterior circulation of the pig

High flow bypass surgery can be a last resort procedure for patients suffering from complex neurovascular pathology. Temporary occlusion of a large recipient artery in these patients could result in debilitating neurological deficits. The ELANA arteriotomy system is intended to create an intracranial anastomosis in a non-occlusive manner. It requires considerable skills form the neurosurgeon to connect the donor graft to the recipient vessel with micro sutures, most often at the intracranial ICA [7, 8]. This procedure, even in experienced hands, is time consuming and takes a minimum of 60 min for each anastomosis to be completed. Therefore, we developed a sutureless, mechanical anastomotic connection device, the SELANA slide (SEsl). The SEsl was proven feasible in laboratory and animal experiments [9–11] but failed during human application on the basis of formation of a pseudo aneurysm due to traction during insertion and translation in the ICA [12]. After careful evaluation of this clinical case, critical changes were made to the design to create an improved device: the SELANA clip (SEcl). The SEcl has several critical advantages over the prior static design of the SEsl. A spring was added to the back of the device which connects the ring and insertion pins (Fig. 1a). Opening and closing of the clip enables smooth insertion, translation, optimized visualization, and subsequently improved hemostasis (Fig. 2b,c). In previous studies [1, 2], we determined the acute feasibility of this technique in a laboratory setting and an experimental rabbit model. We concluded that the SEcl anastomosis was superior in application time and not inferior in flap retrieval rate, hemostasis, and burst pressure compared with the conventional ELANA technique in a rabbit model.

In the present study, we aim to determine the long-term non-inferiority and safety of the SEcl technique compared with historical data of the conventional ELANA anastomosis technique [6, 7, 13]. We assess application times, flap retrieval rates, hemostasis, patency, flow and microscopical endothelialization, and remodeling. In accordance with earlier ELANA research, this study is the next logical next step in the path towards clinical application.

## Materials and methods

This study was conducted from December 2011 until November 2012 and was approved by the animal experimentation committee of the Utrecht University, Utrecht, the Netherlands. All protocols on animals were according to the regulations of Good Laboratory Practice. The implantation study was approved by the Animal Experiments Commission (DEC) of the Utrecht University under DEC registration number 2011.II.06.112.

### Animals

For this study, we used 18 female Dutch Landrace pigs (mean weight 31.0 kg ± 3.5). Preoperatively, the pigs had a normal diet and 1 daily dose of 100 mg calcium carbasalate from 6 days before surgery until sacrifice. For this study, we have chosen the porcine model as it is the most similar to the human conditions in weight, organ similarity, and vascular physiology. During the experiments, we encountered 5 terminal complications due to non-anastomosis–related factors, e.g., laryngospasm, which are known complications [3, 4]. Pigs are difficult to intubate on the basis of a long narrow mouth and an angulation into the trachea. This difficulty increases the number of attempts needed to successfully intubate the animal, which results in the emergence of larynchospasm [5]. Because no determinations on outcome measures could be made, these animals were excluded from our study.

### Anesthesia

Pre-medication was intravenous (iv) administration of midazolam (0.7 mg/kg) and sufentanil (0.007 mg/kg). Subsequently, anesthesia was induced by thiopental (4 mg/kg) and atropine (0.02 mg/kg), and also 1 dose of amoxicillin/clavulanic acid (10 mg/kg) was administered. After positioning in supine position and intubation, a continuous saline infusion was started (300 mL/h) containing sufentanil (0.0133 mg/kg) and midazolam (1 mg/kg).

### Procedure

All procedures were performed by the first author (BdB). During the study, we performed surgery on 18 female Dutch Landrace pigs in six different survival groups: 4 h, 2 days, 1 week, 2 weeks, 3 weeks, and 6 months. In each animal, one bypass was made. Each bypass consisted of two separate end-to-side SEcl anastomoses, which were interconnected by a traditional hand sutured end-to-end anastomosis.

First, the pig was positioned in a supine position under general anesthesia with the neck in deflection. After a midline incision in the neck, the right common carotid artery (CCA) was harvested over approximately 10 cm. The vessel was then flushed using a heparin solution and divided into two equal parts (2 × 5 cm length). At one end of each 5-cm section, a SEcl was attached by guiding the donor through the ring and then folding the donor back over the ring (Fig. 1a,b). Subsequently, the SELANA catheter 2.0, a modified version of the standard ELANA laser catheter, was inserted. A fixation clip was then positioned over the donor and catheter, so a fixed entity was formed (Figs. 1b and 2a). Thereafter, with a 90° angle between the applier and the clip, the SEcl/donor complex was inserted in the recipient artery (Fig. 1c–e). The improved visibility over the SEsl technique and the insertion pins reduces the risk of mispositioning of the SEcl (Fig. 2b–d).

After correct positioning of the SEcl/donor complex on the recipient left CCA, we used 3 times 16.7 mJ to lase the arteriotomies (Fig. 1f).

After lasing and retrieval of the flap (Fig. 1 g,h), 5000 IE of heparin was administered in both anastomoses. When the flap was not retrieved together with the SELANA catheter 2.0, an escape procedure was performed to retrieve the flap. Two options were used. In one option, two temporary clips were placed proximal and distal to the SEcl anastomosis. Then, the SEcl was opened to allow retrieval of the flap by forceps. In the other option, we again ceased circulation by temporary clip, after which a longitudinal incision was made in the donor to access the anastomosis and manually retrieve the flap using forceps. Thereafter, the incision was closed by standing sutures.

Following the flap retrieval, both donor vessels were temporally clipped to prevent back flow from the CCA and were then end-to-end hand sutured with Prolene 8.0 (Ethicon Endo-Surgery (Europe) GmbH) to create an interposition jump-bypass. After flow measurement in the bypass, the left CCA was occluded between the anastomoses, so the anterior circulation was dependent on the bypass (Fig. 2f). The flow in the bypass was measured before wound closure.

Post-operatively, the animals were housed in a separate stable for one week to secure optimal wound healing. For 3 days the animals were scored on neurological function, and body temperature was measured. When no complications occurred within the first 7 days after surgery, the animals were housed in groups. Certified animal caretaker personnel performed monitoring of animal health and welfare according to our standardized and approved protocols.

Post-operative patency of the bypass was determined by angiography before termination in all animals, except in the 4-hour survival group in which we used the intra operative flow measurements to determine patency. In the animals in the 6-month survival group, an interim angiography was performed after three weeks of the initial bypass surgery. If the bypass was occluded, the animal was terminated.

Per survival group, one anastomosis was analyzed using electron microscopy and one by histology, distributed ad random, to determine endothelialization and remodeling. Hereto, the anastomoses were stored in formalin immediately after removal from the pig.

For histology, the anastomoses were embedded in plastic. After a 7-day fixation, coupes were made using a diamond saw and stained with hematoxylin and eosin.

Complications rates, flow values, endothelialization, and bypass patency were compared with historical series on laboratory animals [6, 13].

### Application time

The application time of the SEcl technique was defined as the start of the fixation of the donor and SELANA catheter 2.0 to the SEcl until the retrieval of the flap. The manual retrieval times are included in the total times.

### Flow measurements

Intraoperative flow was measured using a single-vessel flow meter (Transonic Systems Inc.®, Ithaca, NY, USA). Before removing the right CCA, flow was determined in both CCAs. Subsequently, the flow was measured after removal of the right CCA and also when the bypass was patent. Before sacrifice, the flow in the bypass was assessed for the last time.

### Animal sacrifice

During animal sacrifice, the anesthesia protocol as described previously was used. After flow determination, the left CCA, including the bypass, was dissected. Sacrifice was executed by sodium pentobarbital when the animal was still under general anesthesia.

### Sample size

To optimize reduction in the number of animals needed, we accepted a minimum patency of 80% to define non-inferiority for the SEcl anastomosis compared with the conventional ELANA anastomosis [6]. The sample size calculation, using 80% power, defining an 80% patency in the SEcl anastomoses as the lower border of equivalence and tested one-sided with *α* = 0.05, resulted in minimally 36 SEcl anastomoses. For the calculations, the open-source calculator clincalc.com was used.

### Statistical analysis

Differences in patency, hemostasis, and flap retrieval rate of the SEcl anastomosis and the ELANA anastomosis were performed using the Fisher’s exact test. The differences in application time and flow between the SEcl anastomosis and the ELANA anastomosis were assessed by a non-paired *t* test. For all statistical analyses, the open-source calculator graphpad.com was used. Numbers are stated ± standard deviation (SD) unless otherwise indicated. We regarded a *p* value of smaller than 0.05 as statistically significant.

## Results

A total of 37 SEcl anastomoses were made to create 18 bypasses in 18 animals. In one animal, an added anastomosis was made as an extra anastomosis attempt during an acute experiment as training.

### Application time and flap retrieval

The mean SEcl application time was 15.2 ± 9.6 min for the 37 anastomoses This was significantly faster (*p* < 0.0001, Table 1) than the conventional ELANA anastomosis described by Streefkerk et al. [6], with mean difference of 30.0 min (95% CI 25.3–34.7).

**Table 1:** Comparison between the SEcl and the ELANA techniques

Variable	SEcl^a^	ELANA^b^	*p* value
Number of pigs	18	28	–
Animal weight at surgery (kg ± SD)	31 ± 4	31 ± 4	NS
Attachment time (min ± SD)	15 ± 10	45 ± 9	< 0.0001
Flap retrieval (n/n total)	32/37 (86%)	Unknown	–
Oozing after lasing (n/n total)	2/37 (5%)	14/28 (50%)	< 0.0001
Brisk after lasing (n/n total)	2/37 (5%)	1/56 (2%)	NS
Mean flow at opening (mL/min)	159 ± 36	145 ± 29	NS
Mean flow at 6 months (mL/min)	157 ± 25	169 ± 45	NS
Patency (n/n total)	17/18 (94%)	24/28 (86%)	NS
Complete endothelialization	3 weeks	2 weeks	–

^a^This study

^b^Streefkerk et al. [6]

After lasing with the SELANA catheter 2.0, the flap was successfully retrieved by the catheter in 32 of 37 anastomoses (86%). The flap was not retrieved by the catheter in 5 anastomoses (in 3 bypasses) and was manually extracted in all these procedures by either opening of the SEcl or performing a longitudinal incision in the donor.

### Hemostasis

There was full hemostasis in 33/37 (89%) anastomoses. In 2 anastomoses (5%), there was oozing leakage, which was self-limiting in 1 min. It was superior to the conventional ELANA anastomosis (*p* < 0.0001). In 2 anastomoses (5%), Surgicel was needed to obtain hemostasis, and this was scored as brisk leakage, which was comparable to the ELANA anastomosis (Table 1).

### Patency

**Fig. 3:**
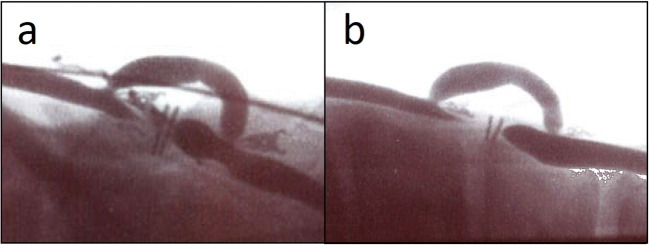
Postoperative angiogram. (**a**) Control angiogram at 3 weeks. Flow is directed from left to right. The carotid artery donor is ectatic between the two anastomoses. (**b**) Angiogram just before termination at 6 months. Mean flow at termination was 157 mL/min. Flow is directed from left to right. The carotid artery donor is ectatic between the two anastomoses

One animal did not survive the pre-determined survival time due to a hematoma (non-surviving animal). No objective determination of the patency could be made. In all other bypasses (*n* = 17), the bypass was patent. The total patency rate was determined as 17 out of 18 bypasses (94%). When a bypass was open at three weeks (Fig. 3a), no occlusions occurred during follow-up after 6 months (Fig. 3b). The 94% patency was above the pre-determined lower limit of non-inferiority of the study design (80%).

### Flow

During surgery, the mean flow in the left CCA before the removal of the right CCA was 193.2 ± 47.0 mL/min (*n* = 18). After removal of the right CCA, the mean flow in the left CCA was 257.7 ± 53.7 mL/min (*n* = 18). The mean flow through the bypasses before closing was 159.6 ± 35.5 mL/min (*n* = 18). At 6 months during termination, the mean flow in the bypasses was 156.5.0 ± 24.7 mL/min (*n* = 6), and this was comparable to the ELANA bypass at 6 months (169 ± 45 mL/min)

### Endothelialization

**Fig. 4:**
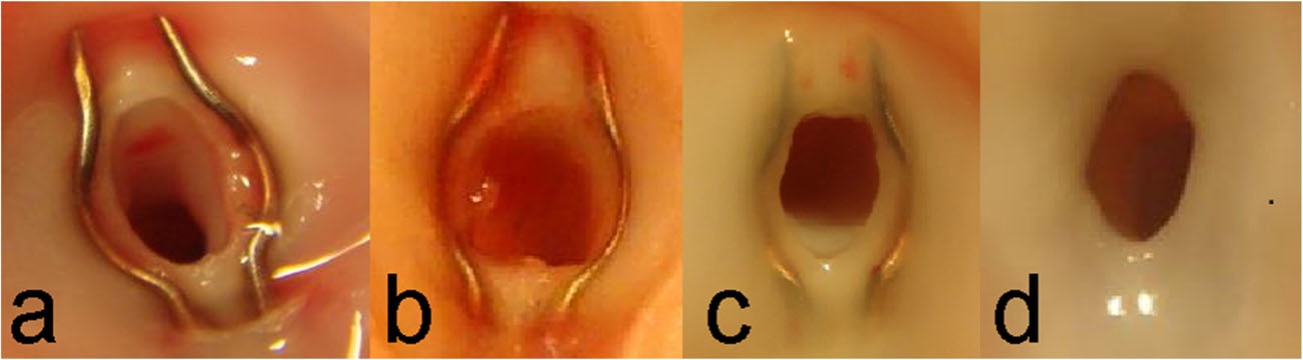
Photographs of anastomoses after termination (view from inside). (**a**) In 2 days survival. No endothelialization of the pins, laser edge, or edge between bypass graft and recipient. (**b**) In 1 week survival. Starting endothelialization over the pins. (**c**) In 3 weeks survival. Complete endothelialization, laser edge is remodeling. Endothelialized pins are still slightly visible. (**d**) In 6 months survival. Complete endothelialization. Arterial remodeling under the ring, the contour of the pins is hardly visible anymore

**Fig. 5:**
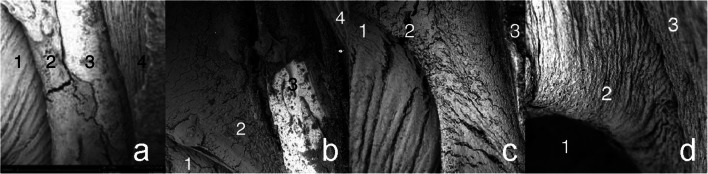
Scanning electron microscopy. 1, Donor graft; 2, laser edge; 3, pin; 4, recipient artery. (**a**) In 2 days: clear demarcation between (from left to right) bypass graft, laser edge, pin, and recipient artery. (**b**) In 1 week: endothelium started to grow over the pin, preceded by activated thrombocytes and fibrin that adhere to the smooth surface of the pin (white/dark gray demarcation left central). (**c**) In 3 weeks: the SELANA clip is completely endothelialized, slightly sharp demarcation of the laser edge. (**d**) In 6 months: complete endothelialization and remodeling of the laser edge

**Fig. 6:**
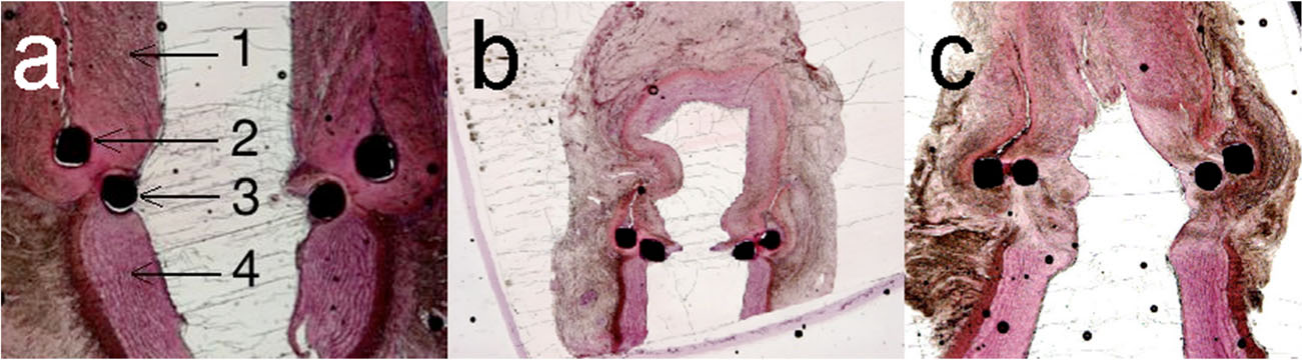
Histology. The anastomosis was embedded in plastic, cut with a diamond saw perpendicular to the recipient CCA, and stained with hematoxylin and eosin. 1, Donor; 2, ring; 3, pins; 4, recipient artery. (**a**) In 1 week, 40× magnification: no endothelialization. (**b**) In 3 weeks, 20× magnification: full endothelialization. (**c**) In 6 months, 20× magnification: a new arterial wall covers the ring and pin

In the second subgroup (2 days after the bypass completion, Figs. 4a and 5a), there was a clear demarcation between bypass graft, laser edge, pin, and recipient artery. The catheter did not damage the intima directly mechanically or indirectly via the laser pulses, and the endothelial cell layer of the donor artery and recipient artery was undamaged. The different layers of the recipient artery (intima, media, and adventitial layer) could clearly be distinguished on the laser edge, which was covered by platelet aggregates and fibrin depositions (Fig. 6). During the first week, an increasing number of activated thrombocytes, with long villus-like protrusions, could be found between bypass graft and laser edge, and over the pin (Figs. 4b, 5b, and 6a). Endothelialization of the pin seemed to originate from the tips and both sites where the recipient arterial wall was penetrated by the pin (Fig. 4).

At 3 weeks (subgroup 5, Figs. 4c, 5c, and 6b), both endothelialization of the pin, as well as endothelialization of the edge between bypass graft and laser edge, was completed. A thin layer of newly formed endothelium totally covered the anastomosis (Fig. 6).

At 6 months (subgroup 6, Figs. 4d, 5d and 6c), the pins were still slightly visible from inside the anastomosis; however, the device is completely covered by a layer of endothelium.

Both macroscopic observations at the time of termination of the animal and excision of the anastomosis, and macroscopic photography, microscopic (SEM), or histological analysis of the tissue samples do not show any abnormal biological effects, like fibrosis/inflammation, tissue degeneration, necrosis, or toxicological effects.

### Biocompatibility

The pins of the SEcl are made of alloyed titanium (ISO 5832-3), which has excellent characteristics as an implantable material (corrosion resistant and biocompatible). Up until three weeks after insertion of the SEcl in the recipient artery, the pins have a surface area inside the lumen. To ensure biocompatibility in the pre-clinical phase, hematological specimens were taken which did not show signs of inflammation or thrombosis (Appendix Table 2). Additionally, the SEcl is fully MR compatible.

### Non-surviving animal

One animal died of a post-operative bleed which can be appointed as a device-related adverse event. The insertion of the proximal clip entity was more troublesome, but the lasing process was without difficulties, and the anastomosis was without leakage. The distal anastomosis showed brisk leakage after initial flawless insertion and lasing. Also, minor leakage was seen at the end-to-end anastomosis along the sutures. After lasing, the bypass was patent but leakage persisted out of the end-to-end anastomosis. SURGICEL® (Ethicon Endo-Surgery (Europe) GmbH) was applied to cease this leakage. During closure of the operating wound, some leakage persisted from the end-to-end anastomosis which we appointed as a surgical error. In accordance with the research protocol, heparin was administered. The obduction revealed a compressing hematoma and no signs of a pseudoaneurysm at one of the SEcl anastomoses.

## Discussion

In this study, we aimed to determine the long-term feasibility of the SEcl anastomosis. In our previous studies, we demonstrated the acute feasibility in a laboratory and rabbit model [1, 2]. Five pre-determined factors were compared with the previous study on the ELANA technique by Streefkerk et al [6].

First, we consider the SEcl superior in application time to the conventional ELANA technique, with mean difference of 30.0 min (95% CI 25.3–34.7). This considerably shortens anesthetic time for patients. Eliminating the need for deeply located microsutures on the ICA and proximal MCA simplifies the anastomotic technique.

Second, the SEcl anastomosis is superior to the ELANA technique in hemostasis directly after lasering (oozing after lasering 5% vs 50%, *p* < 0.0001). In our recent study [1], we opted the need for optimization of the sharpening process of the pins to insure adequate insertion and translation to prevent excessive bleeding and false aneurysm formation. We consider this an ongoing process in which progress is a constant factor. We did encounter suboptimal insertion, though it has to be noted that the wall thickness of the carotid artery of the pig is thicker than the internal carotid artery in a human model (0.3–0.4 mm compared with 0.1–0.2 mm) [11]. This is the reason we lased using 3 times 16.7 mJ compared with 2 times 10 mJ in human setting. Despite our efforts, a compressing hematoma was found to be the cause of death in one of the animals. After meticulous analysis of the case, we concluded it to be a surgical error. In retrospect, we observed brisk leakage from the end-to-end anastomosis and failed to address the possible effects of the hematoma formation. This hematoma was not caused by a pseudoaneurysm formed at the SEcl anastomoses. Therefore, the current sharpness and configuration of the clip device are believed to be sufficient and safe enough to prevent the complication of forming a false aneurysm at the insertion location. In all animals, 5000 IE of heparin was administered. This could have negatively influenced the fatal bleeding that occurred in one animal.

Third, this study showed a 94% (17/18) patency rate of the SEcl anastomosis technique. We consider this a success, though this simplification of the initial ELANA technique still requires sufficient training before it can be applied. The patency rate was comparable to the 86% for the ELANA technique (*p* = 0.64)

Fourth, the flow through the SEcl bypass (157 ± 25 mL/min) was comparable to the flow through the ELANA bypass at 6 months (169 ± 45 mL/min)

Last, this study showed that the endothelialization process of the SEcl anastomosis is completed after approximately 3 weeks, which is 50% longer than the conventional ELANA anastomosis in an identical porcine model. The ELANA anastomosis showed complete endothelialization in approximately 2 weeks [6]. We believe that this difference does not have clinical consequences, whereas the long-term patency of both anastomoses is comparable. After endothelialization, the endothelium remodels to a smooth anastomosis.

Comparing the SEcl in a porcine model to the historical data of the ELANA anastomosis has a distinct drawback of not comparing techniques in exact comparable circumstances. In previous studies on the SELANA slide (SEsl), the same comparison was made [9–11]. After determining the pre-clinical feasibility of the SEsl, the clinical application however failed due to the formation of a pseudoaneurysm [12]. Following extensive evaluation of the clinical case, essential changes were made to the device, which led to the SEcl design. During evaluation of our study results, we determined hemostasis and possible pseudoaneurysm formation as a priority. The death of one of the study animals, based on formation of a hematoma, was not caused by leakage from a SEcl anastomosis, but at the end-to-end anastomosis. By minimizing the friction during insertion and translation into the recipient artery, we believe to have adequately minimized the chance of pseudoaneurysm formation.

Based on the current study results, a human pilot study is the next step in the determination of a safe non-occlusive anastomosis technique.

## Conclusion

The SEcl technique is not inferior to the ELANA technique regarding patency, flow, and endothelialization. On the basis of a significantly shorter application time and superior hemostasis, the SEcl technique could be preferable over the ELANA technique. A pilot study in patients is a logical next step based on our current results.

## Appendix

**Table 2:** Laboratory results

Parameter	Value	Value	Unit	Ref values	Ref values in humans
*Blood chemistry*	*#19*	*#21*			
Urea	2.8	3.6	mmol/L	< 8	3.0–7.5
Creatinine	147	147	μmol/L	< 221	74–120
Glucose (hep)	5.9	4.3	mmol/L	3.3–4.0	3.6–5.6
Na+	140	138	mmol/L	135–150	136–146
K+	3.0	3.3	mmol/L	4.3–6.7	3.8–5.0
Cl−	99	96	mmol/L		99–108
Ca++	2.41	2.36	mmol/L	2.5–3.0	2.2–2.6
Phosphate	2.05	2.11	mmol/L		0.8–1.5
Alk. phosphatase	65	92	U/L		0–120
ALAT	55	89	U/L	8–76	0–45
ASAT	39	39	U/L	12–272	0–35
Bilirubin direct	< 1.7	< 1.7	μmol/L		
Bilirubin total	3.9	2.3	μmol/L	< 3.4	3–21
Gamma-GT	47	45	U/L		0–55
Total protein	56	54	g/L	45–70	60–80
Albumin	36	34	g/L	40–62	35–50
Cholesterol	1.9	1.6	mmol/L		3.5–6.5
Triglycerides	0.15	0.32	mmol/L		0–2
*Hematology*	*#19*	*#21*			
Hb	6.2	6.2	mmol/L	6.2–8.7	8.6–10.7
HcT	0.28	0.27	L/L	0.35–0.45	0.41–0.5
Erythrocytes	5.2	5.2	× 10^12^/L	5–8	4.2–5.5
MCV	53.6	52.6	fL		80–97
MCH	1.2	1.2	fmol		1.75–2.25
MCHC	22.3	22.7	mmol/L		19–23
Leucocytes	8.7	8.9	× 10^9^/L	10–18	4–10
Lymphocytes	5.7	5.5	× 10^9^/L	33–56	0.8–4
Monocytes	0.3	0.3	× 10^9^/L	1–3	0.2–0.8
Eosinophils	0.2	0.2	× 10^9^/L	0–7	0–0.4
Basophiles	0	0.1	× 10^9^/L	0–2	0–0.2
Thrombocytes	204	236	× 10^9^/L		150–450
Prothrombin time	16.2	16.6	s		11.5–14.5
APTT	37.1	40	s		29–39
